# Prevalence of Nicotine and Tobacco Product Use by Sexual Identity, Gender Identity, and Sex Assigned at Birth Among Emerging Adult Tobacco Users in California, United States

**DOI:** 10.1093/ntr/ntad048

**Published:** 2023-03-25

**Authors:** Evan A Krueger, Chenglin Hong, Nicole J Cunningham, Lorree (Katy) Berteau, Luisita Cordero, Elizabeth S C Wu, Ian W Holloway

**Affiliations:** School of Social Work, Tulane University, New Orleans, LA 70112, USA; Department of Social Welfare, Luskin School of Public Affairs, University of California, Los Angeles, Los Angeles, CA 90095, USA; Health Services, Los Angeles LGBT Center, Los Angeles, CA 90028, USA; Prevention and Implementation Sciences Core, Center for AIDS Research, Emory University, Atlanta, GA 30322, USA; Department of Social Welfare, Luskin School of Public Affairs, University of California, Los Angeles, Los Angeles, CA 90095, USA; Department of Social Welfare, Luskin School of Public Affairs, University of California, Los Angeles, Los Angeles, CA 90095, USA; Department of Social Welfare, Luskin School of Public Affairs, University of California, Los Angeles, Los Angeles, CA 90095, USA

## Abstract

**Introduction:**

Sexual and gender minority (SGM) nicotine and tobacco use disparities are well-documented among youth and young adults (YYA), and despite decades of prevention efforts, these disparities stubbornly persist. To better understand tobacco use disparities and craft tailored interventions, tobacco use patterns must be assessed in a contemporary sample of YYA across lines of sexual and gender identity, sex assigned at birth, and tobacco product types.

**Aims and Methods:**

Data were from an online survey of a diverse sample of emerging adult tobacco users (ages 18–29; *N* = 1491) in California, United States (2020–2021). Participants were recruited from various online and in-person locations. Bivariate and adjusted models assessed differences in four nicotine and tobacco use outcomes (past 30-day use of cigarettes, e-cigarettes, other tobacco products, and multiple tobacco product types) across six groups: Cisgender heterosexual males, cisgender heterosexual females, cisgender sexual minority (SM) males, cisgender SM females, transfeminine participants, and transmasculine participants.

**Results:**

Compared to cisgender heterosexual males, both transfeminine (OR = 2.25, 95% confidence intervals (CI) = 1.29 to 4.05) and transmasculine (OR = 1.85, 95% CI = 1.32 to 2.80) participants had higher odds of using cigarettes. Few differences were noted between groups in use of e-cigarettes. Cisgender heterosexual males had higher odds of other tobacco product use, compared to most other groups (eg, cisgender SM males: OR = 0.57, 95% CI = 0.37 to 0.87). Transmasculine participants had higher odds of multiple product use, compared to cisgender heterosexual females. Among multiple product users, transfeminine participants had the highest prevalence of using all three individual product types (35.6%).

**Conclusions:**

Results highlight the need for different tobacco control approaches across sexual and gender identities, sex assigned at birth, and nicotine and tobacco products.

**Implications:**

SGM nicotine and tobacco use disparities remain entrenched, despite concerted efforts to reduce them. The SGM population is heterogeneous and different SGM subgroups may have different needs. This study assessed, among young adult nicotine and tobacco users in California, U.S. patterns of tobacco use across sexual and gender identities, sex assigned at birth, as well as specific tobacco products used—a necessity to craft tailored tobacco control measures. We found patterns of nicotine and tobacco product use across several of these characteristics, highlighting how different prevention and cessation interventions may be needed to meaningfully address SGM nicotine and tobacco use disparities.

## Introduction

Cigarette use has declined considerably among youth and young adults (YYA) in the United States over the past two decades,^1^ due in large part to regulations that limited YYA exposure to cigarette marketing and products (eg, the 1998 Master Settlement Agreement prohibited the use of cartoons such as Joe Camel, which appealed to young audiences, in cigarette advertisements).^2,3^ However, YYA use of alternative nicotine and tobacco products, including e-cigarettes, has increased rapidly in recent years.^4^ For instance, in 2019, 27.5% of U.S. high school students reported using e-cigarettes in the past 30 days, compared to only 1.5% in 2011.^5^ The rapid uptake of alternative nicotine and tobacco products among YYA has created a need for close surveillance and swift regulatory action—especially as use of these products among tobacco-naïve young people may lead to uptake of cigarette smoking and other combustible tobacco use.^6^ To craft effective policies, there is a need to understand contemporary YYA patterns of nicotine and tobacco use across a range of product types, including cigarettes, e-cigarettes, and other nicotine or tobacco products, as well as use of multiple product types.

Nicotine and tobacco use are not evenly distributed across demographic subgroups of YYA. Sexual and gender minority (SGM; eg, lesbian, gay, bisexual, and transgender) YYA use tobacco at disproportionately high rates, compared to their non-SGM peers.^7,8^ For example, in the 2015 Youth Risk Behavior Survey (a U.S. national survey of high school students, grades 9-12; *N* = 14 703), 40.5% of lesbian/gay youth reported past-month any tobacco use, compared to 29.6% of heterosexual youth.^9^ However, the SGM YYA tobacco use literature has been limited in two ways: First, the bulk of extant research has focused on describing SGM cigarette use disparities,^10-12^ though there is growing evidence that SGM YYA also use e-cigarettes and other alternative tobacco products at higher rates than non-SGM youth.^9,11^ For instance, 40.2% of transgender youth in the Population Assessment of Tobacco and Health, wave 3 (2015–2016; *N* = 7772; ages 14–17) reported lifetime use of e-cigarettes, compared to 23.0% of cisgender youth.^13^

Second, existing surveillance studies have not consistently measured the necessary constructs, including sexual (eg, heterosexual, lesbian, gay, and bisexual) and gender (cisgender, transgender, gender non-binary, or non-conforming) identities, and sex assigned at birth (female vs. male), and/or have been underpowered to assess patterns of use with sufficient granularity.^14,15^ The SGM populations are heterogenous, and studies are only just beginning to document how nicotine and tobacco use patterns across SGM subgroups, with some subgroups (eg, sexual minority [SM] women and gender minority populations) commonly experiencing the deepest disparities in use.^9,16–18^ Furthermore, SGM nicotine and tobacco use disparities commonly vary by sex assigned at birth (ie, between those assigned female vs. male at birth); among SM populations, tobacco use disparities are commonly found between SM and heterosexual females, with relatively few such disparities found between SM and heterosexual males.^9,16,19^ There is a noted absence, however, of studies examining sex differences in nicotine and tobacco product use among gender minority YYA (ie, between transmasculine [gender minority participants assigned female at birth] and transfeminine [gender minority participants assigned male at birth] YYA).^20^ To meaningfully improve tobacco control initiatives and policies so that they are responsive to all members of the SGM population, there remains a vital need to first identify patterns of nicotine and tobacco product use across sexual and gender identity, and by sex at birth.

### Study Aims

This study aims to compare prevalence rates of nicotine and tobacco product use across six groups of emerging adult (ages 18–29) tobacco users in California, United States: Cisgender heterosexual males and females, cisgender SM males and females, and transfeminine and transmasculine YYA.

## Methods

### Participants and Procedures

Between March 2020 and August 2021, young adults (ages 18–29) from California, United States were recruited to participate in an online survey designed to compare nicotine and tobacco use behaviors between SGM and non-SGM young adults. Potential participants were recruited using many online and in-person strategies. First, young adults utilizing clinical and/or housing services at the Los Angeles LGBT Center who reported tobacco use in the electronic medical record were invited to participate in the study. Second, a direct mailer campaign was conducted, whereby recruitment materials were mailed to all addresses in neighborhoods that had higher concentrations of SGM residents and/or venues that catered to SGM clientele. These neighborhoods were identified using internet searches for “top LGBTQ-friendly neighborhoods in California,” and included West Hollywood, in Los Angeles; Long Beach in Los Angeles; and the Castro District in San Francisco. Third, the study was advertised via online mainstream social media and gay dating apps, with paid posts placed on Grindr, Adam4Adam, Facebook, Craigslist, Instagram, and Reddit. Posts were also shared by study staff with internet communities, such as SGM-related Facebook groups and relevant Reddit sub-forums (“sub-Reddits”). Study staff also reached out to individuals, nonprofit organizations, universities, healthcare clinics, and other organizations online via email and direct messages on Instagram. “Influencers” (people with a large number of social media followers) were also recruited to advertise the study through social media posts. Fourth, study materials were also distributed to local venues, such as bars, clubs, and coffee shops. Study materials were also handed out to attendees at a local West Hollywood Pride event. Fifth, both SGM and non-SGM participants were recruited from an existing online panel via the survey company Qualtrics.

Regardless of recruitment source, potential participants completed an online screener to determine eligibility for the study. Eligible participants had to (1) reside in California, United States, (2) read and write in English or Spanish, (3) be between the ages of 18 and 29, (4) have used a nicotine or tobacco product within the last 30 days, and (5) provide informed consent. Study procedures were approved by the Univeristy of California, Los Angeles Office of the Human Research Protection Program (OHRPP).

### Variables

#### Nicotine and Tobacco Use Outcomes

Participants were asked to select all the products they had used in the past 30 days from a list. From this list, four main outcome variables were created, including any use of: (1) cigarettes (manufactured cigarettes; hand-rolled cigarettes; clove cigarettes bidis/beedis, kreteks), (2) e-cigarettes (JUUL, [other] e-cigarettes, e-hookah, e-bowl, e-pen, e-pipe, and e-cigars), and (3) other nicotine or tobacco products (cigars, cigarillos, little filtered cigars, or cheroots; regular pipes full of tobacco; hookah; smokeless tobacco: Snus, chewing tobacco, snuff, or dip; dissolvable tobacco), and (4) use of multiple nicotine or tobacco products (any combination of two or more of the above categories).

#### Sexual Identity, Gender Identity, and Sex Assigned at Birth

Participants were asked to report their current sexual orientation (“which of the following best describes your current sexual orientation?”: Straight or heterosexual, lesbian, gay, bisexual, queer, pansexual, same-gender loving, asexual, and sexual orientation not listed here). Participants were categorized as heterosexual or SM (any identity other than “straight or heterosexual”). Participants also reported their current gender identity (“If you had to choose only one of the following terms, which best describes your current gender identity?”: Cisgender male/man, cisgender female/woman, transgender male/man, transgender female/woman, genderqueer/ gender non-binary/ gender non-conforming/ gender fluid, gender identity not listed here). Participants were categorized as either cisgender (cisgender male/man, cisgender female/woman) or gender minorities (any identity other than cisgender male/man or cisgender female/woman). Finally, participants reported their sex assigned at birth (“What sex were you assigned at birth, on your original birth certificate?”) as either male or female. Based on participants’ responses to these three items, participants were assigned to one of six categories: Cisgender heterosexual males, cisgender heterosexual females, cisgender SM males, cisgender SM females, transfeminine participants (gender minority participants assigned male at birth), and transmasculine participants (gender minority participants assigned female at birth).

It should be noted that gender identities occur on a spectrum, and many participants endorsed non-binary and diverse identities beyond “cisgender” and “transgender.” We use the terms “transfeminine” and “transmasculine” as umbrella terms to capture the diversity of identities participants utilized, while stratifying by sex assigned at birth, as was done for cisgender heterosexual and cisgender SM participants. In our sample, transfeminine participants identified as transgender female/woman (44%), genderqueer/gender non-binary/ gender non-conforming/fluid (52%), and with a gender identity not listed (4%). Transmasculine participants identified as transgender male/man (23%), genderqueer/gender non-binary/ gender non-conforming/fluid (72%), and with a gender identity not listed (5%).

#### Covariates

Covariates include age, race and ethnicity (white, Asian, black/African American, Hispanic/Latino or Spanish origin, multiracial, and other [included American Indian/Alaska Native, Native Hawaiian/Pacific Islander, Middle Eastern/North African, and a race not listed here]), country of birth (the United States, outside the United States), educational attainment (less than a general education diploma/high school diploma, general education diploma/high school diploma, some college/Associate’s degree, Bachelor’s degree, or higher), employment (full-time, part-time, self-employed, other, student, and unemployed; those indicating more than one employment status were assigned the highest-ranking option according to the order listed here), and housing stability (unstable, stable; coded as unstable if any of the following were reported alone or in combination: on the street, in a car, in an abandoned building, in a park, or a place that is not a house, apartment, shelter, or other housing; substance abuse treatment center or sober living; in a shelter; in a group home facility), and marital status (married/living with a partner, single, and divorced/separated/widowed/other).

### Data Analysis

Sociodemographic characteristics were first calculated across the six groups, and differences were tested using chi-square (categorical variables) and ANOVA (continuous variables) tests. Next, prevalence of each of the four main outcomes (any use of: cigarettes, e-cigarettes, other nicotine or tobacco products, and use of multiple nicotine or tobacco products) were calculated, and compared across the six groups. Multiple logistic regressions then estimated group differences in each of the four main outcomes, adjusting for sociodemographic covariates. Results are reported as adjusted odds ratios (aORs) with corresponding 95% confidence intervals (CI). Cisgender heterosexual males served as the referent group, though post hoc pairwise comparisons, using Benjamini–Hochberg corrected *p*-values, also tested differences in the outcomes between cisgender heterosexual females, cisgender SM females, and transmasculine participants, and between cisgender SM males and transfeminine participants. Next, among users of multiple nicotine or tobacco product types, the specific combinations of product types used were calculated and compared across the six groups.

## Results

### Sample and Sociodemographic Characteristics

In total, 7626 participants were screened for the study from across the various recruitment strategies, and 1491 eligible participants (868 SGM, 623 non-SGM) participants were enrolled in the study.

As shown in Table 1, race/ethnicity varied across the groups (*p* < .001), with for instance, 33.9% of cisgender heterosexual males and 41.8% of cisgender SM males identifying as White, and 10.9% of cisgender heterosexual males and 31.9% of transmasculine participants identifying as multiracial. Country of birth also varied across groups (*p* = .011), for instance, 91.7% of cisgender heterosexual males and 82.1% of transfeminine participants were born in the United States. Employment status varied across groups (*p* < .001), for instance, 38.7% of cisgender heterosexual males and 22.3% of cisgender SM females were employed full-time. The groups also differed with regard to housing stability (*p* < .001). For instance, 14.3% of transfeminine participants and 3.5% of cisgender heterosexual males reported being unstably housed. Marital status varied across the groups (*p* < .001), with for instance, 35.6% of cisgender SM females and 17.9% of transfeminine participants reporting they were married or living with a partner. There were also small age differences across groups (*p* = .007), with cisgender SM females and transfeminine participants being the youngest on average (both means* *= 22.9), and cisgender SM males being the oldest on average (mean = 23.7). No differences were noted in educational attainment (*p* = .113).

**Table 1.** Sociodemographic Characteristics of the Sample

	Full sample	Cisgender heterosexual males	Cisgender heterosexual females	Cisgender SM males	Cisgender SM females	Transfeminine participants	Transmasculine participants	*p*-Value
	*N* = 1491 (100.0%)	*N* = 313 (21.0%)	*N* = 310 (20.8%)	*N* = 208 (14.0%)	*N* = 385 (25.8%)	*N* = 84 (5.6%)	*N* = 191 (12.8%)	
Race and ethnicity (%)								<.001
White	537 (36.0)	106 (33.9)	103 (33.2)	87 (41.8)	154 (40.0)	29 (34.5)	58 (30.4)	
Asian	234 (15.7)	72 (23.0)	60 (19.4)	29 (13.9)	46 (11.9)	6 (7.1)	21 (11.0)	
Black	85 (5.7)	22 (7.0)	10 (3.2)	12 (5.8)	19 (4.9)	8 (9.5)	14 (7.3)	
Hispanic/Latinx	329 (22.1)	66 (21.1)	79 (25.5)	46 (22.1)	87 (22.6)	16 (19.0)	35 (18.3)	
Other	50 (3.4)	13 (4.2)	15 (4.8)	6 (2.9)	10 (2.6)	4 (4.8)	2 (1.0)	
Multiracial	256 (17.2)	34 (10.9)	43 (13.9)	28 (13.5)	69 (17.9)	21 (25.0)	61 (31.9)	
Country of birth (%)								.011
Outside the United States	26 (8.3)	26 (8.4)	26 (8.4)	21 (10.1)	21 (5.5)	15 (17.9)	17 (8.9)	
United States	287 (91.7)	284 (91.6)	284 (91.6)	187 (89.9)	364 (94.5)	69 (82.1)	174 (91.1)	
Educational attainment (%)								.113
Less than GED or HS diploma	55 (3.7)	9 (2.9)	12 (3.9)	4 (1.9)	16 (4.2)	3 (3.6)	11 (5.8)	
GED or HS diploma	304 (20.4)	80 (25.6)	64 (20.6)	26 (12.5)	73 (19.0)	22 (26.2)	39 (20.4)	
Some college/associate’s degree	722 (48.4)	143 (45.7)	145 (46.8)	114 (54.8)	194 (50.4)	36 (42.9)	90 (47.1)	
Bachelor’s degree or higher	410 (27.5)	81 (25.9)	89 (28.7)	64 (30.8)	102 (26.5)	23 (27.4)	51 (26.7)	
Employment status (%)								<.001
Full time	440 (29.5)	121 (38.7)	87 (28.1)	69 (33.2)	86 (22.3)	21 (25.0)	56 (29.3)	
Part time	339 (22.7)	53 (16.9)	64 (20.6)	48 (23.1)	100 (26.0)	24 (28.6)	50 (26.2)	
Self-employed	94 (6.3)	26 (8.3)	11 (3.5)	12 (5.8)	28 (7.3)	8 (9.5)	9 (4.7)	
Other	29 (1.9)	3 (1.0)	12 (3.9)	0 (0.0)	10 (2.6)	0 (0.0)	4 (2.1)	
Student	314 (21.1)	54 (17.3)	79 (25.5)	37 (17.8)	94 (24.4)	11 (13.1)	39 (20.4)	
Unemployed	275 (18.4)	56 (17.9)	57 (18.4)	42 (20.2)	67 (17.4)	20 (23.8)	33 (17.3)	
Housing Stability (%)								<.001
Unstable	84 (5.6)	11 (3.5)	15 (4.8)	10 (4.8)	17 (4.4)	12 (14.3)	19 (9.9)	
Stable	1407 (94.4)	302 (96.5)	295 (95.2)	198 (95.2)	368 (95.6)	72 (85.7)	172 (90.1)	
Marital status (%)								<.001
Married or living with partner	431 (28.9)	76 (24.3)	102 (32.9)	38 (18.3)	137 (35.6)	15 (17.9)	63 (33.0)	
Single	1030 (69.1)	231 (73.8)	201 (64.8)	163 (78.4)	243 (63.1)	66 (78.6)	126 (66.0)	
Divorced or separated or widowed or other	30 (2.0)	6 (1.9)	7 (2.3)	7 (3.4)	5 (1.3)	3 (3.6)	2 (1.0)	
Age (mean)	23.2							

SM = Sexual Minority; GED = General Education Diploma; HS = High School.

### Group-Level Differences in Nicotine and Tobacco Product Use

**Figure 1.**
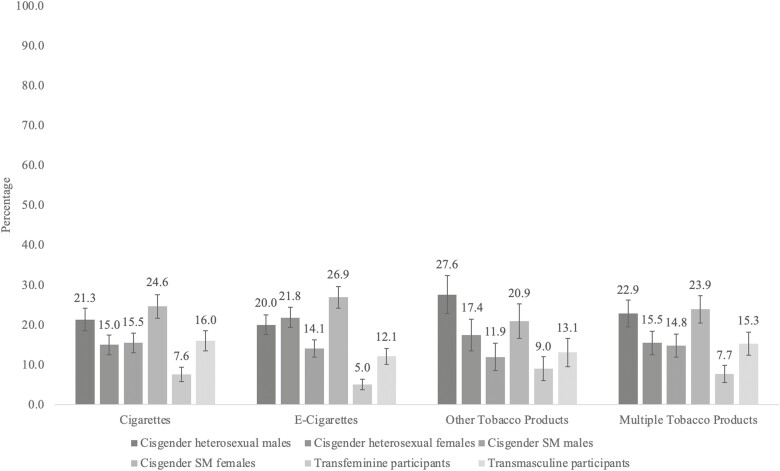
Prevalence of Nicotine and Tobacco Use Outcomes by Sexual Orientation and Gender Identity. Notes. “Other tobacco products” included cigars, cigarillos, little filtered cigars, or cheroots; regular pipes full of tobacco; hookah; smokeless tobacco: Snus, chewing tobacco, snuff, or dip; dissolvable tobacco

Prevalence of past 30-day nicotine or tobacco use is presented separately across the six groups in Figure 1. Transfeminine participants reported the lowest prevalence (7.6%, 5.0%, and 7.7%), and cisgender SM females reported the highest prevalence (24.6%, 26.9%, and 23.9%) of past 30-day cigarette, e-cigarette, and multiple product type use, respectively. While transfeminine participants reported the lowest prevalence of other tobacco use (9.0%), straight cisgender males reported the highest prevalence (27.6%).

Group-level differences in past 30-day nicotine or tobacco use, adjusted for covariates, are presented in Table 2. Compared to cisgender heterosexual males, cisgender heterosexual females had lower odds of cigarette use (aOR = 0.56, 95% CI = 0.40 to 0.80), while transfeminine participants (aOR = 2.25, 95% CI = 1.29 to 4.05) and transmasculine participants (aOR = 1.85, 95% CI = 1.23 to 2.80) both had higher odds of cigarette use. Post hoc pairwise comparisons also indicated that both cisgender SM females and transmasculine participants had higher odds of cigarette use than cisgender heterosexual females, and transmasculine participants had higher odds of cigarette use than cisgender SM females (all Benjamin–Hochberg *p* < .05).

**Table 2.** Past 30-Day Use of Various Nicotine and Tobacco Products, Multiple Logistic Regressions

	Cigarette use		E-cigarette use	Other tobacco product use		Use of multiple tobacco products	
	aOR (95 % CI)		aOR (95 % CI)	aOR (95 % CI)		aOR (95 % CI)	
Sexual orientation and gender identity		a, b, c			d		b
Cisgender heterosexual males	Ref		Ref	Ref		Ref	
Cisgender heterosexual females	0.56 (0.40, 0.80)**		1.35 (0.93, 1.96)	0.58 (0.40, 0.85)**		0.58 (0.41, 0.82)**	
Cisgender SM males	1.25 (0.86, 1.84)		1.19 (0.80, 1.78)	0.57 (0.37, 0.87)**		0.95 (0.66, 1.37)	
Cisgender SM females	0.96 (0.69, 1.33)		1.26 (0.89, 1.79)	0.54 (0.37, 0.78)**		0.79 (0.57, 1.08)	
Transfeminine participants	2.25 (1.29, 4.05)**		0.85 (0.49, 1.47)	1.18 (0.69, 1.99)		1.46 (0.88, 2.43)	
Transmasculine participants	1.85 (1.23, 2.80)*		0.92 (0.61, 1.39)	0.64 (0.41, 0.98)*		1.12 (0.76, 1.63)	

All models controlled for race and ethnicity, country of birth, educational attainment, employment status, housing stability, marital status, and age. Superscripts denote pairwise differences (*p* < .05) identified between (a) cisgender heterosexual females and cisgender sexual minority (SM) females, (b) cisgender heterosexual females and transmasculine participants, (c) cisgender SM females and transmasculine participants, and (d) cisgender SM males and transfeminine participants. Significance tests were corrected for multiple comparisons using the Benjamini–Hochberg correction method. ***p < .001, **p < .01, *p < .05. CI = confidence intervals.

No differences were noted in past 30-day e-cigarette use across the six groups.

Compared to cisgender heterosexual males, cisgender heterosexual females (aOR = 0.58, 95% CI = 0.40 to 0.85), cisgender SM males (aOR = 0.57, 95% CI = 0.37 to 0.87), cisgender SM females (aOR = 0.54, 95% CI = 0.37 to 0.78), and transmasculine participants (aOR = 0.64, 95% CI = 0.41-0.98) all reported lower odds of past 30-day use of other nicotine or tobacco products. Compared to cisgender SM males, transfeminine participants had higher odds of other nicotine or tobacco product use (Benjamin–Hochberg *p* < .05).

Cisgender heterosexual females had lower odds of using multiple nicotine or tobacco products, compared to cisgender heterosexual males (aOR = 0.58, 95% CI = 0.41 to 0.82). In post hoc pairwise comparisons, transmasculine participants had higher odds of using multiple products, compared to cisgender heterosexual females (Benjamin–Hochberg *p* < .05).

Among those using multiple tobacco products, the specific combinations of product types used are shown in Table 3, separately by group. Over half (52.4%) of those using multiple product types reported using both cigarettes and e-cigarettes, though a larger proportion of cisgender SM males reported using this combination of product use (60.5%), compared to transfeminine participants (42.2%; *p* = .031). Overall, 12.4% of the sample reported using cigarettes and other tobacco products, and 13.4% reported using e-cigarettes and other tobacco products, with no group-level differences noted for these combinations of products. However, 21.8% of those using multiple products reported using products from all three categories, with 35.6% of transfeminine participants reporting this combination of use, compared to 13.3% of cisgender heterosexual females (*p* < .001).

**Table 3.** Combinations of Tobacco and Nicotine Products Used Among Users of More Than One Product (*N* = 582)

	Full sample (N = 582)	Cisgender heterosexual males (*N* = 133)	Cisgender heterosexual females (*N* = 90)	Cisgender SM males (*N* = 86)	Cisgender SM females (*N* = 139)	Transfeminine participants (*N* = 45)	Transmasculine participants (*N* = 89)	*p*-Value
Tobacco products used								
Cigarettes + e-cigarettes (%)	305 (52.4)	59 (44.4)	45 (50.0)	52 (60.5)	82 (59.0)	19 (42.2)	48 (53.9)	.031
Cigarettes + other tobacco products (%)	72 (12.4)	21 (15.8)	13 (14.4)	9 (10.5)	11 (7.9)	7 (15.6)	11 (12.4)	.127
E-cigarettes + other tobacco products (%)	78 (13.4)	22 (16.5)	20 (22.2)	6 (7.0)	18 (13.0)	3 (6.7)	9 (10.1)	.285
Cigarettes, e-cigarettes, and other tobacco products (%)	127 (21.8)	31 (23.3)	12 (13.3)	19 (22.1)	28 (20.1)	16 (35.6)	21 (23.6)	<.001

SM = sexual minority

## Discussion

Despite declining rates of cigarette use among the general population of emerging adults,^1^ there still exist wide-ranging SGM disparities in cigarette use. Our data reflect this trend, with several cigarette smoking disparities noted across sexual and gender identities; cisgender SM females reported higher odds of smoking cigarettes in the prior 30 days versus their cisgender heterosexual counterparts, and both transmasculine and transfeminine participants reported higher odds of past 30-day cigarette smoking versus their cisgender heterosexual and cisgender SM counterparts (though the estimate obtained for transfeminine participants had a relatively wide confidence interval).

While many contemporary youth perceive cigarettes as being more harmful than other tobacco products^21,22^—likely because of successful smoking prevention campaigns—ample research has shown that SGM young people smoke cigarettes due, at least in large part, to discrimination and other forms of minority stress.^23,24^ It is thus possible that SGM young people have a greater willingness to engage in behaviors perceived as harmful (ie, cigarette smoking) to cope with minority stress.^25,26^ Relatedly, recent studies have shown that while SGM smokers indeed associate cigarette use with poorer health, some may also view smoking as a necessary act of social resistance (ie, intentionally engaging in stigmatized behaviors such as smoking as a way of “pushing back” against social stigma, including homophobia and transphobia).^27,28^ Tobacco companies, attuned to the stigmatization and legal and social restrictions faced by members of the SGM community, have been successful in framing tobacco use as a personal right and freedom.^29–31^ Conversely, by stigmatizing cigarette smoking, prevention and cessation interventions may inadvertently re-stigmatize SGM people, contributing to continued cigarette use. Thus, to mitigate SGM cigarette smoking disparities, prevention and cessation interventions may need to acknowledge SGM young peoples’ experiences with minority stress and the myriad reasons SGM use tobacco products, without stigmatizing the act of cigarette smoking itself.

Comparatively fewer group differences were noted in past 30-day e-cigarette use (though it should be noted that cisgender heterosexual [21.8%] and cisgender SM [26.9%] females had the highest bivariate prevalence estimates for e-cigarette use, highlighting the increasing public health concern paid to tobacco use among young females^32^). The absence of a disparity in e-cigarette use may reflect the high rates of e-cigarette use among young people across the sexual and gender identity spectrum. Youth e-cigarette use has been described as a new epidemic,^33^ with almost 23% of middle and high schoolers reporting e-cigarette use in 2020.^34^ As such, efforts to reduce e-cigarette use may need to target both SGM and non-SGM youth, though additional research is needed to understand whether SGM YYA’s reasons for using e-cigarettes differ from their non-SGM peers. While not comparative, a recent qualitative study of SGM males and gender-diverse youth found that psychological/ physical distress was the top reason participants reported for smoking or vaping, endorsed by 38.7% of participants. Other commonly cited reasons included desires to conform to group norms and/or to build new social networks.^35^

The prevalence of other tobacco product use was highest among cisgender heterosexual males. This finding is in line with prior studies showing that (cisgender heterosexual) males use other tobacco products, such as smokeless tobacco, at higher rates than females.^36^ These tobacco products have historically been marketed towards males, with marketing efforts historically emphasizing masculinity as part of “brand identity.”^37^ Thus, efforts to reduce use of non-cigarette and e-cigarette tobacco products may need to target cisgender heterosexual males, addressing themes of masculinity. However, it will be important for future studies with sufficient analytic power to assess SGM differences in use of individual tobacco product types included in this category (eg, cigars vs. snus vs. hookah).

Transmasculine participants had higher odds of past 30-day use of multiple tobacco product types, compared to cisgender heterosexual females. Furthermore, while transfeminine participants reported the lowest prevalence of each of the four primary outcomes, among those using multiple product types, transfeminine participants had the highest prevalence of using all three product types. Thus, future tobacco cessation interventions tailored to this group should consequently consider multi-product use. Moreover, there is a dearth of research regarding tobacco use patterns among gender non-conforming and non-binary young adults. Future research efforts should focus explicitly on this population, making efforts to disentangle differences across gender minority subgroups (eg, between transgender and non-binary young people).

### Limitations

This study has limitations. First, data were derived from a convenience sample of emerging adults from California, United States. While efforts were made to recruit a diverse sample, these findings may not be representative of emerging adults across the state, in the United States more broadly, or in other countries. Second, given that recent tobacco/nicotine use was an inclusion criterion for participation in the study, the tobacco and nicotine use prevalence estimates obtained (for all groups) are expected to be higher than those of the general population. Furthermore, given that SGM YYA utilizes tobacco at higher rates than non-SGM YYA on average,^7,8^ it might be expected that the SGM sample obtained more closely mirrors the wider SGM YYA population, as compared to non-SGM participants, who may be less representative of the wider non-SGM YYA population. Thus, given the sampling strategy employed, it is important to restate that these analyses compared prevalence estimates of tobacco and nicotine product use across groups of *recent nicotine or tobacco users*. Third, the measures used to assess sexual and gender identities were developed with U.S. emerging adults in mind, and alternate measures may be more appropriate for assessing these constructs in other countries and contexts. Fourth, to increase power, several decisions were made to collapse key analytic variables for analysis. Specifically, all SM identities were collapsed into a single analytic group. Gender minority participants were also collapsed into a single group, regardless of the sexual identity they endorsed. This approach will have masked any differences in tobacco use between gender minority heterosexual and gender minority SM emerging adults. However, by doing so, we were able to compare tobacco use differences between gender minority participants assigned male at birth and those assigned female at birth. In addition, given low prevalence of other tobacco product use within the sample, all non-cigarette and non-e-cigarette tobacco products were collapsed into a single analytic category. Additional research is needed to identify differences in use of specific tobacco products within this category.

## Conclusion

There exist broad disparities in tobacco product use among SGM YYA. However, tobacco use research must not assess SGM people as a monolithic population. The magnitudes of—and likely, the mechanisms driving—SGM tobacco use disparities vary widely across sexual and gender identities, sex assigned a birth, as well as by tobacco product type used. While cigarette use disparities remain strongly entrenched across most SGM subgroups, multiple tobacco use is also of particular public health concern. To meaningfully address SGM tobacco use disparities, tobacco control efforts must consider the unique populations and factors contributing to tobacco use among diverse SGM populations.

## Supplementary Material

A Contributorship Form detailing each author’s specific involvement with this content, as well as any supplementary data, are available online at https://academic.oup.com/ntr.

## Data Availability

The data underlying this article will be shared on reasonable request to the study’s senior author.
